# Supplementary material for: How Precisely Can Easily Accessible Variables Predict Achilles and Patellar Tendon Forces during Running?
Source: Sensors (Basel). 2021 Nov 8;21(21):7418. doi: 10.3390/s21217418 (PMC8587337; doi:10.3390/s21217418)
Supplement: Supplementary file 1 [file sensors-21-07418-s001.zip › sensors-1428532-supplementary.pdf]

Supplementary material: How precisely can outdoor measurable variables predict structure-specific loads in the Achilles and patellar tendon during running?

Table S1: shows the descriptive statistics of the anthropometrics

Variables	mean	sd	min	max
Bodyweight (kg)	82	11	60	102
Body height (cm)	180	7	165	191
Knee height (cm)	50	3	44	56
Ankle height (cm)	8	1	6	9
Shoe sole height (cm)	3	1	1	4

Table S2: shows the descriptive statistics and the effect of running speed on tendon force and impulse across running speed

	**All**				**10km/h**				**12km/h**				**14km/h**			
Variables	mean	sd	min	max	mean	sd	min	max	mean	sd	min	max	mean	sd	min	max
**Training load**																
Ground contact time length (ms)	252	25	199	308	268	24	218	308	251	22	206	289	238	21	199	272
Vertical oscilation (mm)	95	13	68	132	92	13	68	124	96	12	78	132	97	13	75	132
Cadence (step/min)	83	4	74	91	82	4	75	88	83	4	74	89	85	4	77	91
Stride length (cm)	1173	126	911	1443	1053	91	911	1256	1168	83	944	1310	1291	68	1186	1443
**Patellar tendon**																
Peak force (N)	5268	915	2639	7616	5102	960	2639	7616	5361	880	3285	7462	5337	890	3221	7143
Impulse force (kN/stride)	152.7	33.3	61.4	273.0	161.3	35.4	75.2	273.0	155.7	31.6	83.9	233.7	141.2	29.6	61.4	21.5
**Knee joint moment**																
Peak joint Moment (Nm)	174	47	65	280	154	40	69	243	171	46	65	265	196	46	82	280
Peak joint Moment / BW (Nm/kg)	2,16	0,5	0,8	3,4	1,9	0,4	0,8	3	2	0,5	0,8	3,3	2,4	0,5	1	3,4
**Achilles tendon**																
Peak force (N)	5150	1528	2185	9493	4562	1388	2185	8232	5165	1476	2288	9493	5709	1506	2604	9380
Impulse force (kN/stride)	112.4	40.6	36.6	222.0	112.5	44.5	41.8	221.4	113.8	39.8	36.6	222.0	111.4	37.5	39.0	202.1
**Ankle joint moment**																
Moment (N*m)	-193	108	-368	-15	-218	85	-368	-15	-207	107	-365	-18	-158	118	-368	-19
Moment / BW (N*m/kg)	-2,43	1,3	-0,2	-4,6	-2,7	1	-0,2	-4,6	-2,6	1,3	-0,3	-4,6	-2	1,5	-0,3	-4,6
